# Expecting to integrate additional information improves averaging of experience

**DOI:** 10.1038/s41598-024-67369-z

**Published:** 2024-07-18

**Authors:** Guy Grinfeld, Marius Usher, Nira Liberman

**Affiliations:** 1 School of Psychological Sciences, Tel Aviv University, Tel-Aviv, Israel; 2 Sagol School of Neuroscience, Tel Aviv University, Tel-Aviv, Israel; 3 International Faculty, Key Profile Area II: Behavioral Economic Engineering and Social Cognition, University of Cologne, Cologne, Germany

**Keywords:** Abstraction, Average extraction, Information integration, Learning from experience, Social learning, Verbal communication, Psychology, Human behaviour

## Abstract

Humans learn both directly, from own experience, and via social communication, from the experience of others. They also often integrate these two sources of knowledge to make predictions and choices. We hypothesized that when faced with the need to integrate communicated information into personal experience, people would represent the average of experienced exemplars with greater accuracy. In two experiments, Mturk users estimated the mean of consecutively and rapidly presented number sequences that represented bonuses ostensibly paid by different providers on a crowdsource platform. Participants who expected integrating these values with verbal information about possible change in bonuses were more accurate in extracting the means of the values compared to participants who did not have such expectation. While our study focused on socially communicated information, the observed effect may potentially extend to other forms of information integration. We suggest that expected integration of experience with additional information facilitates an abstract representation of personal experiences.

## Introduction

How long does it take you to get to work? What is the average electricity bill in your household? What is the average cost of a dinner in your city? People often face the need to accurately estimate the average of multiple experiences to plan action (e.g., decide when to leave to work), compare alternatives (e.g., which city offers cheaper dining), and evaluate outcomes (e.g., were my efforts to conserve electricity effective?). The average glosses over the specifics of the exemplars, and represents them by a single, relatively stable value. Its usefulness, however, depends on the accuracy with which it is estimated.

Both classic theories^1–3^ and modern models of value integration^4–6^ suggest that people can estimate averages of values efficiently and accurately. This research also found, however, that accuracy of averaging may be distorted by giving extra weight to specific exemplars. Extra weight can be given to exemplars that are encountered early (i.e., a primacy effect)^2,7,8^, and/or late (i.e., a recency effect)^9–11^. Extra weight might be given also to extreme or vivid exemplars^12–15^, and even to just random exemplars in the flow of experience. All of these would result in noisier, less accurate estimation of the average. These lines of study and theorizing are consistent with the possibility that people estimate the average more accurately if they pay less attention to individual exemplars, and instead focus on the common phenomenon that those exemplars represent.

In this paper, we investigate whether the accuracy with which people estimate the average of their experiences improves when they have to incorporate information that comes from out of the scope of their experience and treats their flow of experiences as a single phenomenon. Consider, for example, a new sales associate at a retail company trying to gauge their own sales performance over their first few months. They receive daily sales reports detailing the number of products sold and total revenue generated. Some days stand out due to exceptionally high or low sales, which can bias their perception of overall performance. However, if the associate knows they will receive periodic market analysis reports from the corporate office, including industry benchmarks and comparisons with other stores, they might focus more on identifying general trends in their sales data. Would anticipating such external information encourage them to form a more accurate average representation of their performance and reduce the bias that stems from paying more attention to individual, memorable sales days? We would like to suggest that the answer is positive.

We often face a need to estimate our experience as a whole and integrate it with information that comes from the experience of other people. For example, we may need to form a prediction of how long our drive home would take upon hearing of an accident on the way; estimate how much our expenditures would increase once we learn about anticipated inflation; or estimate how hot would next Easter be in view of global warming.

What might improve people’s averaging ability is the need to integrate information that comes from outside of their immediate experience. This external information could take various forms, including verbal communication, one’s own past experiences, or even anticipated future experiences. Here we focus on integrating communicated information, as communication is an important source of information that one often needs to integrate with own experience. While the underlying mechanisms may potentially apply to integrating information from other sources as well, our study focuses specifically on socially communicated information.

Indeed, the ability to integrate socially communicated information into one’s own experience is an important developmental milestone^16^, and such integration has been postulated by central psychological theories of social learning^17,18^, and cultural transmission^19^. Yet, the question of how such integration affects the way one’s own experience is represented has, to the best of our knowledge, never been examined. Our studies take a step toward answering this question, suggesting that it facilitates a focus on the gist (as opposed to a focus on specific exemplars) of a stream of experiences.

## The current experiments

Our prediction regarding more accurate averaging follows in a straightforward way from the notions that what gets in the way of accurate averaging is attending to exemplars, and that an outside perspective on the entire stream of experience reduces such attention.

To test this prediction, we used a variant of the value-psychophysics paradigm in which participants extract the average value from series of consecutively and rapidly presented numbers^9,13,20–23^.

We hypothesized that expecting to integrate one’s own experience with information that comes from outside of the scope of that experience would enhance the accuracy with which the average is extracted. We asked participants in a crowdsource platform to estimate the average of fast-paced numbers that represented the bonuses that different providers on that platform ostensibly paid to their participants. In the experience-only condition, participants only estimated the average. In the anticipating-integration condition, after estimating the average participants read about the provider’s plan to change bonuses and attempted to predict what the experimenter would pay next. The sequences of numbers and the task of estimating their average was the same in both conditions. The only difference was the presence (vs. absence) of an expectation to integrate the experienced numbers with communicated information. We predicted that the estimation of sequences’ averages would be more accurate when participants expect such communication.

## Study 1

In this study, MTurk participants observed series of numbers that represented bonuses that different providers ostensibly paid. In one block, they only estimated the given average bonus by each provider. In the other block, after estimating the provider’s average bonus, participants received information about the providers’ future plans to increase, decrease, or not change the bonuses, and then participants predicted the next bonus the provider would give.

This manipulation includes a social component, as the information about the provider’s intentions to change or maintain the bonus rate conveys the plans of another person (the provider), plans that are relevant to the participant’s task and that represent information that is external to the participants’ experience. Specifically, the provider’s intentions were aimed to capture information that cannot be directly experienced exemplar by exemplar, and instead are communicated as a verbal summary. We thought that this framing of the averaging task should resonate with MTurk users, who are motivated to accurately evaluate the compensation that different providers offer on the platform, and often share this information and the bonuses they received in dedicated communities^24^.

### Method

#### Ethics information

The reported studies adhered to the ethical guidelines for research involving human participants. The Institutional Review Board (IRB) at Tel-Aviv University approved the study protocol (reference #0000262-1) prior to data collection. Informed consent was obtained from all participants before they took part in the research.

#### Participants

Thirty MTurk users from the US (16 women, 14 men; *M*_age_ = 37.50, *SD* = 9.57) took part in this study in return for $3 (plus a bonus of $0.75 for accuracy). We excluded one participant whose performance accuracy was below threshold (their correlation between estimated and actual means of samples in either block, *r*_(40)_ < 0.305, was below significance level). The sample size was predetermined to detect a medium effect size in a within-participants design.

#### Materials

The study was programmed using the Lab.js package for online studies^25^. Participants completed it remotely using their laptop or desktop computer. Upon entering the study, a prompt message asked participants to enter full screen mode.

We presented to each participant 42 sequences, each with eight numbers between 1 and 99. Numbers in each sequence were sampled from Gaussian distributions (*μ* = 50 + k, *σ* = 20, k ~ U (− 25, 25)) with no successive repetition (in case two identical numbers were sampled successively, the entire sequence was shuffled). An additional sequence of the same length was used in a practice trial.

#### Procedure

On the first screen, we asked participants to carefully read a consent form that informed them about the duration of the study and the payment for completing it. Within the form, we also asked participants to enter the third letter of the word “study” into a comment box at the bottom of the page. Only those who correctly completed this attention check and provided informed consent were permitted to proceed to the study itself. A similar attention screening procedure was employed in Study 2.

On the next screen, participants read “Some HITs on MTurk pay workers bonuses. These bonuses vary depending on various task characteristics, but also depend on the requesters themselves. Can you estimate the average bonus a particular requester pays?”.

Participants then read:

*“Imagine a requester who has been offering a series of different HITs. Workers who participated in them reported the bonuses that they got. We will show you the reported bonus sums, one by one, and ask you to determine the average bonus for that requester.”*

Participants who began with the experience-only block did not receive any additional instructions, but those who began with the anticipating-integration block read in addition:

*“We will then give you information about the requester’s future plans, and ask you to predict the bonus that this requester would pay on next HIT.”*

**Figure 1:**
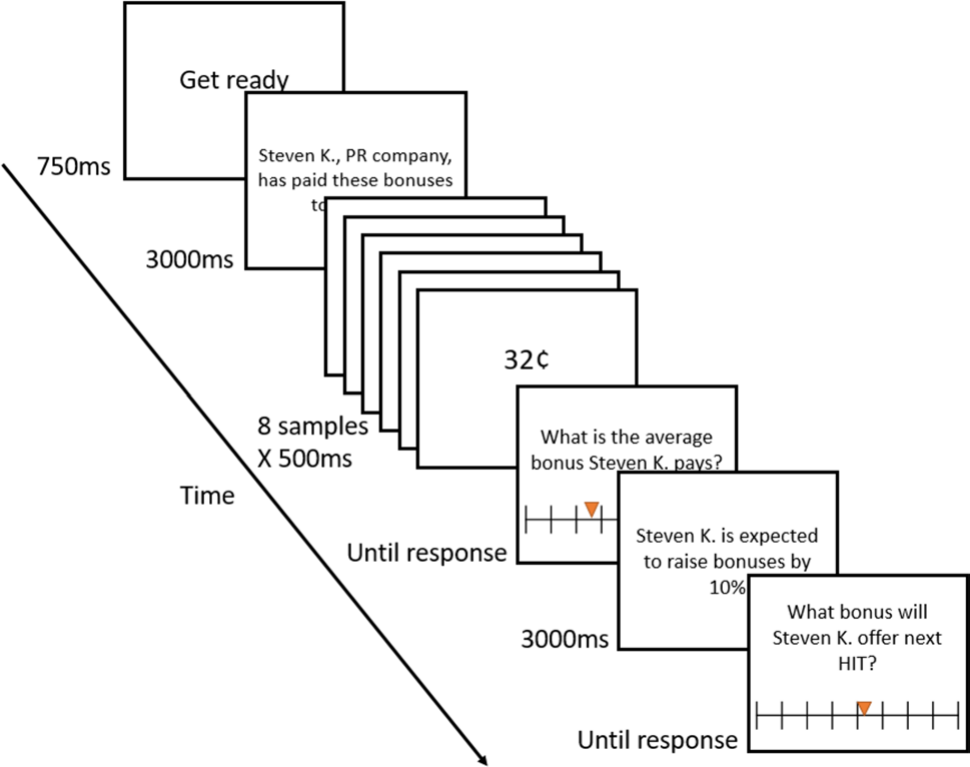
An experimental trial sequence in the anticipating-integration block. Trials in the experience-only block ended after the first response of estimating the sequence’s mean.

Participants completed a practice trial, and then 42 test trails. Each trial began with a ‘get ready’ message (750 ms), followed by a name and affiliation of an experimenter (3000 ms), and a sequence of eight numbers, each presented for 500 ms (Fig. 1). Participants then indicated on an analogue slider scale ranging from 1 to 100 the average bonus the provider paid. Trials in the experience-only block ended at this point. Trials in the anticipating-integration block continued then to present on the next screen information according to which the provider was expected to either increase the bonuses by 7–12%, decrease the bonuses by 7–12%, or not change the bonuses (3000 ms). Participants then predicted the bonus that the provider would pay in their next experiment.

After completing 42 trials, participants proceeded to the next block. They read *“We will now move to a different group of requesters. To clearly distinguish it from the previous group, the bonuses will now appear in a different color. Your task will be slightly different, too.”* Participants who started with the experience-only block read: *“Now, you will not only estimate the average bonus, but also learn additional information and predict a future bonus. After you estimate the average bonus, we will give you information about the requester’s future plans, and ask you to predict the bonus that this requester would pay on the next HIT.”*

Participants who started with the anticipating-integration block read: *“Now, you will only estimate the average bonus. We will show you the reported bonus sums, one by one, and ask you to determine the average bonus for that requester.”*

The second block consisted of one practice trial that was followed by 42 experimental trials. Importantly, the sequences in the second block were the same as in the first, but their order within the block was shuffled. The order of conditions was counterbalanced between participants.

### Results

**Figure 2:**
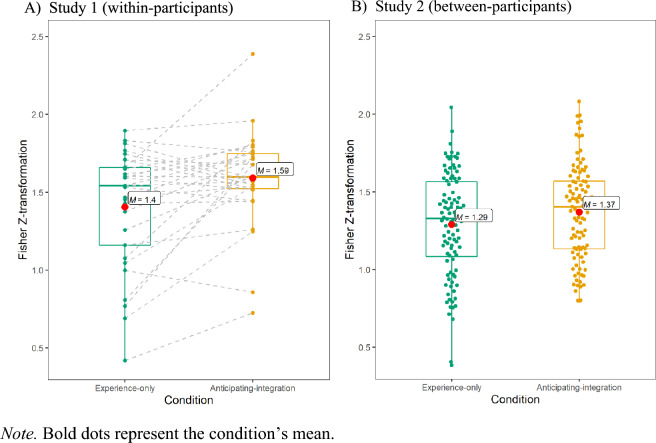
Z-transformed correlations between estimated and actual means.

We used two measures of accuracy: first, we computed the correlation between each participant’s estimates and the actual average across the 42 trials in each block. A paired t-test on the Fisher Z-transformed correlations showed that, as predicted, participants estimated averages more accurately in the anticipating-integration block (*zʹ* = 1.59, *SD* = 0.19) than in the experience-only block (*zʹ* = 1.40, *SD* = 0.19), *t*(28) = 3.01, *p* = 0.006, *g* = 0.54 (Fig. 2A).

The second measure of accuracy was the *RMSE* between estimations and the actual means in each block:

$$RMSE = \sqrt{\frac{\sum {(estimation - actual \,mean)}^{2}}{42}}$$

A paired t-test on the RMSE showed lower root mean deviation (i.e., higher accuracy) in the in the anticipating-integration block (*M* = 7.60, *SD* = 2.24) than in the experience-only block (*M* = 8.73, *SD* = 2.72), *t*(28) = 2.52, *p* = 0.018, *g* = 0.46.

## Study 2

In Study 1, the anticipating-integration condition involved a subsequent prediction, such that trials in this condition involved two tasks, whereas in the experience-only condition trials involved only one task. If the second task distracted participants or took up their cognitive resources, then the results of Study 1 present particularly strong evidence in support of the beneficial effect of anticipating communicated information, as instead of interfering with averaging, it actually enhanced its accuracy. An alternative view, however, could be that because participants in the anticipating-integration condition had to make a subsequent prediction, they held the values in memory or paid more attention to the numbers when observing them, which was also conducive to more accurate averaging.

Study 2 addresses this concern by modifying the experience-only condition to also include a prediction phase. In both conditions, participants were informed about bonuses and had two similar tasks: first, they were asked to estimate the average bonus rate of a provider, and second, they were asked to predict the next bonus. The key difference between the conditions was the presence versus absence of the expectation to integrate the experienced numbers with communicated information about the provider’s intentions. Importantly, our primary dependent variable remained the accuracy of averaging the experienced numbers, which was assessed before participants received any additional information or made predictions about future bonuses.

### Method

#### Participants

Two hundred and two MTurk users from the US (112 women, 92 men; *M*_age_ = 41.31, *SD* = 12.54) took part in this study in return for $2 (plus a bonus of $0.75 for accuracy). Based on our pre-registered criterion, we excluded two participants whose performance was below threshold (their correlation between estimated and actual means of samples, *r*(48) < 0.322, was below a one-tailed significance test with α = 0.01). This sample was sufficient to detect a medium effect size.

#### Materials and procedure

This study used the same materials as Study 1 except that participants had three practice trials, and 48 experimental trials, and the numbers of each sequence were drawn from a Gaussian distribution in which σ = 25. In addition, this study employed a between-participants design.

The procedure was similar to Study 1 except that in the experience-only condition participants read that their task had two stages: first estimate the average bonus a provider gives based on eight samples, and then predict what bonus this provider would pay in their next HIT. To make this condition as similar as possible to the anticipating-integration condition, after estimating the average, participants waited for 3000 ms facing a screen that said: “What would be this experimenter’s next bonus?”.

### Results

A paired one-tailed t-test on the Fisher Z-transformed correlations showed that, as predicted, participants estimated the averages more accurately in the anticipating-integration condition (*zʹ* = 1.37, *SD* = 0.08) than in the experience-only condition (*zʹ* = 1.29, *SD* = 0.11), *t*_*welch*_(198.05) = 1.79, *p* = 0.037, *g* = 0.25 (Fig. 2B).

A paired one-tailed t-test on the RMSE between each participant’s estimations and actual means found lower root mean deviation (i.e., higher accuracy) in the anticipating-integration condition (*M* = 8.75, *SD* = 2.74) than in the experience-only condition (*M* = 9.65, *SD* = 2.89), *t*_*welch*_(199.01) = 2.26, *p* = 0.012, *g* = 0.32.

### Exploratory analyses

As noted above, what often stands in the way of accurate averaging is giving extra weight to some of the samples (rather than weighting them equally). Moreover, it has been found that when extracting the average from fast-paced sequences of numerals, people often exhibit primacy effects^8^, and/or recency effects^9^. To check whether these effects were indeed more pronounced in the experience-only condition than in the anticipating-integration condition, we fitted to each participant in each condition a standardized linear regression of their estimations based on the average of the four samples in extreme temporal positions (i.e., first two and last two presented samples, samples at Places 1, 2, 7 and 8) and the average of the four samples in middle temporal positions (i.e., at Places 3, 4, 5, and 6). We then used the fitted coefficients to check whether estimations in the experience-only condition, compared to those in the anticipating-integration condition, gave more weight to samples in extreme positions relative to samples in middle positions.

**Figure 3:**
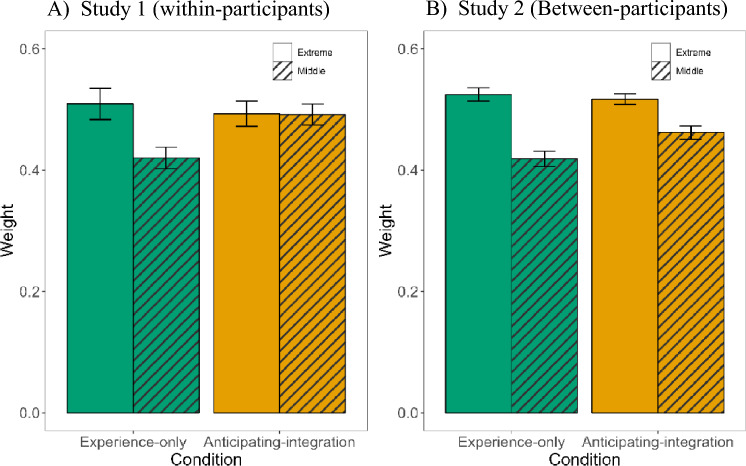
Weight of samples in the extreme and middle places in the estimated averages. Coefficients of samples in extreme places (1, 2, 7, 8 in the sequence) and of samples in middle places (3, 4, 5, 6 in the sequence) in predicting estimated averages of eight samples. Error bars indicate ± 1 SE from the mean. (**A**) Results of Study 1 suggest more weight to samples in extreme positions (compared to samples in middle locations) in the experience-only condition but not in the anticipating-integration condition. (**B**) Results of Study 2 suggest more weight to samples in extreme positions (compared to samples in middle locations) in the experience-only condition more than in the anticipating-integration condition.

In Study 1, we ran a 2 × 2 ANOVA on the coefficients, with experimental condition (experience-only, anticipating-integration) and sample position (extreme, middle) as within-participants variables. A significant interaction, *F*(1, 28) = 5.07, *p* = 0.032, *η*_p_^2^ = 0.15, showed that in the experience-only condition, the coefficients of samples in extreme positions (*M* = 0.509, *SD* = 0.138) were higher than the coefficients of samples in middle positions (*M* = 0.420, *SD* = 0.095), *t*(28) = 2.55, *p* = 0.033 (Bonferroni-adjusted comparison). In contrast, in the anticipating-integration condition, the coefficients of samples in extreme positions (*M* = 0.493, *SD* = 0.112) did not differ from the coefficients of samples in middle positions (*M* = 0.492, *SD* = 0.094), *t*(28) = 0.04, *p* = 0.966 (Fig. 3A).

In Study 2 we conducted a similar analysis, except that experimental condition was a between-participant factor. We found a marginally significant interaction, F(1, 200) = 3.69, p = 0.056, *η*_*p*_^*2*^ = 0.02. Bonferroni-adjusted comparisons indicated that in the experience-only condition, the coefficients of the samples in extreme positions (*M* = 0.53, *SD* = 0.11) were higher than the coefficients of the samples in middle positions (*M* = 0.42, *SD* = 0.13), *t*(200) = 5.62, *p* < 0.001. In the anticipating-integration condition a similar, although somewhat less extreme difference emerged (*M*_*extreme positions*_ = 0.52, *SD* = 0.09; *M*_middle positions_ = 0.46, *SD* = 0.11, *t*(200) = 2.95, *p* = 0.007, Fig. 3B).

In the Supplementary materials we report additional modeling analyses that are commonly used within the value psychophysics tradition^26,27^.

## Discussion

We used the value-psychophysics paradigms to test how the accuracy with which one averages their own experiences is affected by anticipating integrating them with information that comes from other people. Participants estimated the average bonus paid by different providers in a crowdsource platform. In two studies we found that when participants anticipated receiving information on these providers and using it to predict future bonuses, they were more accurate in extracting the average of the experienced values. Importantly, Study 2 addressed the alternative explanation that the improved averaging accuracy could be driven solely by the understanding that accurate mean estimation will aid in performing an additional, subsequent task (rather than only one task of estimating the average). Since both conditions in Study 2 involved estimating the average and predicting the next bonus, the effect cannot be attributed to the mere presence of a second task.

In our paradigm, each value represented a benefit paid by a specific provider on a specific occasion. What sometimes stands in the way of accurate averaging is giving extra weight to some exemplars, such as the first or the last exemplars. We found that participants who had to integrate their experience with information from out of their experience (more than those who did not have to perform such integration) exhibited less pronounced primacy and recency effects, and instead assigned more equal weight to samples in extreme and middle positions. We should emphasize, however, that primacy and recency are not the only factors that might distort accurate averaging. Future studies might examine whether anticipated integration also reduces the impact of outliers (the tendency to give more weight to samples that are extreme relative to the distribution) as well as the impact of other types of salience (e.g., the tendency to give more weight to samples to which we attend longer).

Although we found that anticipating the integration of information from other people enhances the accuracy of averaging, this effect may not be limited to socially communicated information. As we mentioned in the introduction, what might improve people’s averaging ability is the need to integrate information that comes from outside their immediate experience. This external information can take various forms, such as verbal communication from others (as was the case in our studies) as well as considering one’s own experience from outside of the current context (e.g., one’s experiences from the past or even experiences that one anticipates having in the future). Expecting to integrate information from a second, future episode, for example, might trigger one to process currently experienced information more holistically, in preparation for integrating it with experiences of one’s future self. It is interesting to note, in the current context, that research has shown that people relate to their future selves similarly to how they relate to others^28^, suggesting that the effect we observed could extend beyond social communication to encompass integration of current experiences with those of a future self.

In our studies, the social information participants received was about the intentions of different providers, which contrasts with the numerical samples they experienced. While this manipulation includes a social component, it may not be as salient or direct as other social manipulations that have been used in the literature. Future studies might employ more explicit social transmission manipulations, and examine, for example, whether averaging of one’s own experiences is more accurate when participants work in a team whose members share experiences compared to working alone.

Participants in our study experienced samples of payments represented as numerals. It is important to note in this respect that perceiving numeric values resembles other perception processes. Indeed, research has found that even very quick presentations of number symbols trigger number-related activation of the same cortical regions that are activated by analog, non-symbolic values such as size or brightness^29,30^. Also, studies conducted within the value-psychophysics framework found that the values represented by numbers are averaged in a manner similar to non-symbolic aggregations of objects^5,31^. It will be interesting to extend our theorizing beyond numerals to richer, more detailed experiences. For example, would reading professional literature make physicians represent more accurately the average of values that they learned from personal observation in the clinic?

In our studies socially communicated information was introduced after the experience, but our predictions extend to situations where this order is reversed^32^, in which case the question would be how socially communicated verbal priors affect experience. We might predict that regardless of whether such priors create assimilation or contract^33^, they would enhance the accuracy of estimating the mean of one’s experience.

An average is an abstract representation of the exemplars that it summarizes. Just like an abstract category (e.g., dogs) represents different exemplars (e.g., poodle, Airedale-Terrier, Chihuahua) by glossing over how they are different from each other, a mean represents different instances (the exemplars over which the mean was computed) by glossing over their variability^34^. Most relevant to the notion that averaging is an instance of abstraction is a recent set of studies^26^ which demonstrated that participants estimated the average of numerical values and of facial expressions with greater accuracy after being induced with an abstract mindset than after being induced with a concrete mindset (by repeatedly generating abstract goals for performing an action vs. concrete means to perform it, respectively). The current studies add to this earlier work by suggesting that an abstract perspective on one’s own experience is induced by a need to integrate it with information that comes out of the scope of the experience and is communicated by others.

One might contend that when we integrate our experience with communicated information, we consider it from a socially distal perspective^35^. If a mean is an abstraction over experienced exemplars^34^, then our findings are consistent with the notion that psychological distancing facilitates the generation of abstract mental representations, which has been proposed in the framework of Construal Level Theory^36^. This framework would further predict that other ways of increasing psychological distancing would facilitate averaging of own experience. For example, averaging of own experiences would be more accurate when integrating it with information that is communicated by a distal other rather than a socially close person, as well as when the communicated information pertains to temporally more distant events.

The benefit of being exposed to other people’s experiences has long been noted. Typically, the benefit is attributed to the new information that such a perspective affords. Our results suggest yet another benefit: integrating others’ experiences might help us summarize our own experience in a more accurate, less noisy way.

### Supplementary Information

Supplementary Information.

## Data Availability

Deidentified data for both studies, analysis code scripts, and materials used in these studies are available on the project’s OSF page at: https://osf.io/7sydr/. Study 1 was not preregistered; the preregistration for study 2 can be accessed on the OSF project page.
