# Supplementary material for: Expecting to integrate additional information improves averaging of experience
Source: Sci Rep. 2024 Jul 18;14:16627. doi: 10.1038/s41598-024-67369-z (PMC11258318; doi:10.1038/s41598-024-67369-z)
Supplement: Supplementary file 1 — Supplementary Information. [file 41598_2024_67369_MOESM1_ESM.docx]

**Supplementary material**

**Computational modeling**

We fitted the data to several computational models that were used for modeling the value-psychophysics paradigm (Hadar et al., 2020; Rosenbaum et al., 2021; Tsetsos et al., 2012). The first model served as a baseline, assuming normative averaging using equal weighs, constant bias (b_0_) and noise ($\epsilon$) (Table 1). The second model added a third parameter (b_1_) that reflected the extent to which estimations regressed to the midpoint of the scale. The third model introduced to the first model a leak parameter (-1 $\leq\lambda\leq1)$. Negative values indicate a primacy effect, exponentially decreasing weights of later samples. Positive values of this parameter indicate a recency effect, exponentially increasing weighting of later samples. The fourth model added to the third model the parameter that estimated the regression to the midpoint of the scale. Note that $\lambda$ can account to either primacy or recency, but not both effects within a participant.

We fitted the models to the data of each participant in both studies and aggregated the AIC to compare their fit (Table 1). In both studies the fourth model, which includes a leak parameter and a regression parameter, had the best fit.

*Table 1*. Model Comparison

| # | Model | Study 1  Aggregated AIC | Study 2  Aggregated AIC |
| --- | --- | --- | --- |
| 1 | ${y_{j}=b}_{0}+\frac{\sum_{i=1}^{N} x_{i}}{N}+ \epsilon$ | 16,537 | 68,432 |
| 2 | ${y_{j}=b}_{0}+ b_{1}*50+\left( 1-b_{1} \right)*\frac{\sum_{i=1}^{N} x_{i}}{N}+ \epsilon$ | 16,275 | 67,732 |
| 3 | $y_{j}=b_{0}+ \frac{\sum_{i=1}^{N} {\left( 1-\lambda\right)^{N-i}x}_{i}}{\sum_{i=1}^{N} \left( 1-\lambda\right)^{N-i}}+ \epsilon$ | 16,511 | 68,360 |
| 4 | ${y_{j}=b}_{0}+ b_{1}*50+\left( 1-b_{1} \right)*\frac{\sum_{i=1}^{N} {\left( 1-\lambda\right)^{N-i}x}_{i}}{\sum_{i=1}^{N} \left( 1-\lambda\right)^{N-i}}+ \epsilon$ | **16,262** | **67,629** |

*Note.* AIC differences higher than 10 are considered decisive evidence in favor of the model with the lower numerical value. In all models we assume that $\epsilon\sim\mathcal{N}(0, \sigma).$

Next, we used the fitted parameters of each participant to the fourth model to compare the estimations between the anticipating-integration and the experience-only conditions.

In Study 1 estimations in the experience-only condition regressed more to the mid-point of the scale (*M*_b1_ = 0.17, *SD* = 0.18), than did estimations in the anticipating-integration condition (*M*_b1_ = 0.11, *SD* = 0.13), *t*(28) = 2.17, *p* = .040, *g* = 0.39. In addition, estimations in the experience-only condition were more noisy (*M_σ_* = 6.87, *SD* = 2.09), compared with estimations in the anticipating-integration condition (*M_σ_* = 5.91, *SD* = 1.34), *t*(28) = 2.80, *p* = .009, *g* = 0.51. We did not find any difference in the bias parameter, *t*(28) = 0.39, *p* = .700, *g* = 0.07, nor in the leak parameter, *t*(28) = 0.48, *p* = .634, *g* = 0.09.

In Study 2 estimations of participants in the experience-only condition were more noisy (*M_σ_* = 8.08, *SD* = 2.42), compared with estimations in the anticipating-integration condition (*M_σ_* = 7.13, *SD* = 2.09), *t_Welch_*(194.7) = 2.97, *p* = .003, *g* = 0.42. We did not find any difference in the other parameters: Bias, *t_Welch_*(199.9) = 0.21, *p* = .830, *g* = 0.06.; regression , *t_Welsh_*(189.3) = 0.44, *p* = .658, *g* = 0.06; leak, *t_Welsh_*(181.4) = 0.70, *p* = .484, *g* = 0.10.
